# Trauma-Induced Coagulopathy: Overview of an Emerging Medical Problem from Pathophysiology to Outcomes

**DOI:** 10.3390/medicines8040016

**Published:** 2021-03-24

**Authors:** Gabriele Savioli, Iride Francesca Ceresa, Luca Caneva, Sebastiano Gerosa, Giovanni Ricevuti

**Affiliations:** 1 Emergency Department, IRCCS Policlinico San Matteo, PhD University of Pavia, 27100 Pavia, Italy; irideceresa@gmail.com (I.F.C.); seba.gerosa@gmail.com (S.G.); 2 Anesthesia and Intensive Care, Fondazione IRCCS Policlinico San Matteo, 27100 Pavia, Italy; l.caneva@smatteo.pv.it; 3 Department of Drug Science, University of Pavia, 27100 Pavia, Italy; giovanni.ricevuti@unipv.it; 4 Saint Camillus International University of Health Sciences, 00152 Rome, Italy

**Keywords:** early coagulopathy of trauma, acute coagulopathy of trauma-shock, trauma-induced coagulopathy, trauma-associated coagulopathy, major trauma, severe trauma, acute traumatic coagulopathy

## Abstract

Coagulopathy induced by major trauma is common, affecting approximately one-third of patients after trauma. It develops independently of iatrogenic, hypothermic, and dilutive causes (such as iatrogenic cause in case of fluid administration), which instead have a pejorative aspect on coagulopathy. Notwithstanding the continuous research conducted over the past decade on Trauma-Induced Coagulopathy (TIC), it remains a life-threatening condition with a significant impact on trauma mortality. We reviewed the current evidence regarding TIC diagnosis and pathophysiological mechanisms and summarized the different iterations of optimal TIC management strategies among which product resuscitation, potential drug administrations, and hemostatis-focused approaches. We have identified areas of ongoing investigation and controversy in TIC management.

## 1. Introduction

Major trauma (MT) is one of the leading causes of mortality and morbidity globally and the leading cause of death in people younger than 40 years. Annually, traumatic injuries cause approximately 6 million deaths globally [1,2,3]. MT is also a frequent cause of hospitalization, as an estimated 24 million patients are hospitalized yearly. This also results in extensive out-of-hospital medical care for approximately 85 million patients worldwide [1,2,3]. Although the problem mainly affects low- and middle-income countries, high-income countries are also affected. For instance, in Europe major trauma is the third-leading cause of death in the general population and the first among younger patients. Traumatic injuries are also one of the main causes of disability, rendering major trauma a pathology with high direct and indirect costs [1,2,3]. Given its impact on mortality, a quick, efficient, and precise identification of bleeding causes, as well as of coagulopathy is of paramount importance for surgical management [4].

MT is an event that results in a single injury or multiple injuries of such magnitude that it constitutes a quoad vitam or quoad valetudinem (in regard to life or health) danger to the patient. Conventionally, trauma is defined as severe when the patient’s injury severity score (ISS) exceeds 15. ISS is an assessment system that assigns a number based on the severity and location of the different injuries caused by trauma. This index was chosen because it displays excellent correlations with mortality, morbidity, the need for hospitalization, and hospital stay. ISS > 15 was selected on the basis of a proven increase in mortality. ISS can only be calculated after the patient has undergone diagnostic investigations, mainly in a hospital setting. To overcome this limitation, it is essential that a potential MT is recognized as soon as possible in the pre-hospital phase, and triage criteria for MT should be implemented (Table 1).

Post-traumatic hemorrhage is the most frequent cause of death in victims of severe trauma, in about 40% of cases. This is caused by two main mechanisms, but they can intertwine and present simultaneously [4,5,6,7,8,9,10,11,12,13,14].

The first mechanism is bleeding caused via direct injury of blood vessels, which involves hemorrhage that is dependent on physiological or anatomic factors. These include the hemodynamic state of the patient, in particular systolic blood pressure, the arterial or venous nature of the affected vessel, and the caliber of the vessel. In cases of injury of large-caliber arterial vessels, we can witness profuse hemorrhage with shock and exitus in an extremely short period, even before the arrival of the rescue crew.

Meanwhile, the second mechanism is secondary bleeding from the development of trauma-induced coagulopathy (TIC), which involves secondary bleeding from a widespread microvascular hemorrhage that is not localized to the site of the trauma. This represents a pathological entity in its own right, and its classification and pathogenesis will be discussed later.

Approximately 30% of patients with MT develop TIC upon arrival to the emergency department (ED). Although it was once believed that TIC begins hours or even days after the traumatic event, it is currently clear that it begins at the moment of trauma. Approximately 40% of trauma deaths result from bleeding, and 10% of these events appear avoidable [4,5,6,7,8,9,10,11,12,13,14].

## 2. Definition

Numerous definitions and terms have been proposed to identify coagulopathy resulting from trauma and describe the specific pathology of trauma-associated coagulopathy, including acute traumatic coagulopathy, early coagulopathy of trauma, acute coagulopathy of trauma shock, TIC, and trauma-associated coagulopathy [6,15,16,17,18].

TIC can be defined as a condition of endogenous hypercoagulation observed in the immediate post-traumatic period, that is, within 1 h of trauma. It is characterized by widespread microvascular hemorrhage opposed to events localized exclusively to the site of trauma [6,15,16,17,18].

## 3. Pathophysiology

Hemostasis is an essential physiological response to wound healing. It is a dynamic homeostatic process balancing pro- and anti-coagulation systems and fibrinolytic and fibrinolysis-inhibitory pathways, and it consists in the interaction between endothelial cells’ walls, platelets, and clotting factors, with the endothelium taking an active part in this homeostatic process, together with several mediators, among which tissue factor pathway inhibitors, endothelial protein C receptors, the endothelial glycocalyx, thrombomodulin, nitric oxide, and tissue plasminogen activator (tPA) [19].

Despite continuing and recent advances in research into MT and the consequent increase in knowledge in the sector, the pathophysiological mechanisms that contribute to the development of TIC remain largely unknown. This is also associated with the multitude of complex systems that interact with each other [18,19,20,21]. A disturbance in hemostasis is induced by activation/dysregulation of the vascular endothelium, coagulation, natural anticoagulants, the pro-fibrinolytic and anti-fibrinolytic systems, and inflammation [19,20,21].

These phenomena are compounded by a number of external factors (such as hemodilution by the administration of crystalloids) and detrimental factors such as hypothermia, hydroelectrolytic imbalance, and acidosis. These detrimental factors are likely to self-feed and depend on both endogenous and exogenous factors [19,20,21].

**Figure 1:**
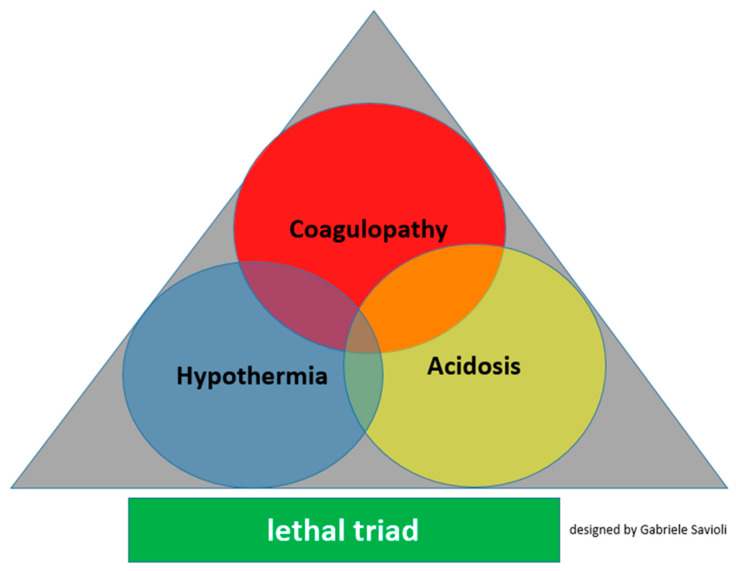
Lethal triad for major trauma.

For years, it was considered that TIC was solely attributable to the dilution of clotting factors caused by substantial fluid administration or massive transfusion, which further complicated the development of acidemia and hypothermia, which, together with TIC, contribute to the formation of the “lethal triad” and thus further aggravate the clinical picture (Figure 1).

Classically, the factors recognized as the only triggers of TIC were hemodilution, hypothermia, and acidemia. Although they are still recognized as TIC triggers, it has been found that TIC develops in the early stages of trauma before any medical intervention and the development of acidemia and hyperthermia. Thus, TIC is dependent on the first phase during the release of mediators by hypoperfused organs and damaged tissues.

With the increasingly greater body of research on the pathophysiological mechanisms of TIC, they have been discovered to be far more complex than initially inferred; additionally, fluid administration has been proven to contribute to the development of intracardiac thrombus without being the main cause, indicating a multifactorial etiopathogenesis.

A distinction can therefore be made between acute traumatic coagulopathy and coagulopathy disease induced by resuscitating maneuvers, which can coexist but possess different mechanisms and temporal phases [19,20,21].

**Figure 2:**
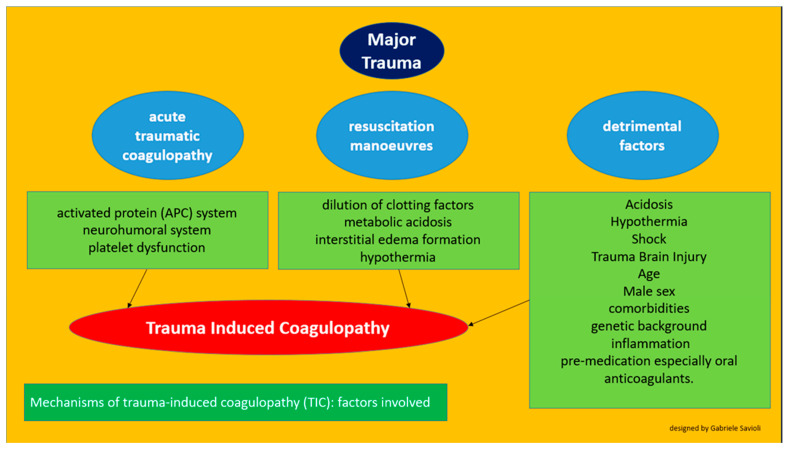
Factors involved in the development of trauma-induced cardiomyopathy.

We can schematically (Figure 2) claim that TIC consists of the following variables:

a pathophysiological process linked to trauma (acute traumatic coagulopathy)

iatrogenic factors (coagulopathy induced by resuscitation maneuvers)

detrimental factors (both iatrogenic and pathophysiological)

### 3.1. Acute Traumatic Hypercoagulability

Injury to the wall of a vessel as a result of trauma can expose subendothelial collagen and activate tissue factors, which provide an adhesion platform for circulating platelets and support the interplay between the cellular and humoral components of the hemostatic system. This pro-coagulant activity is controlled by a counter-regulatory system of natural anticoagulants. The summed effect of these two opposing systems may trigger the coagulatory response at the site of endothelial injury while preventing uncontrolled microvascular thrombosis and tissue hypoperfusion by providing endogenous anticoagulation and fibrinolysis. Parts of the process are interconnected in a complex manner, with thrombin playing a central role by being able to partake in both coagulation and anti-coagulation pathways in addition to interacting with the inflammatory response.

#### 3.1.1. Role of the C Protein

**Figure 3:**
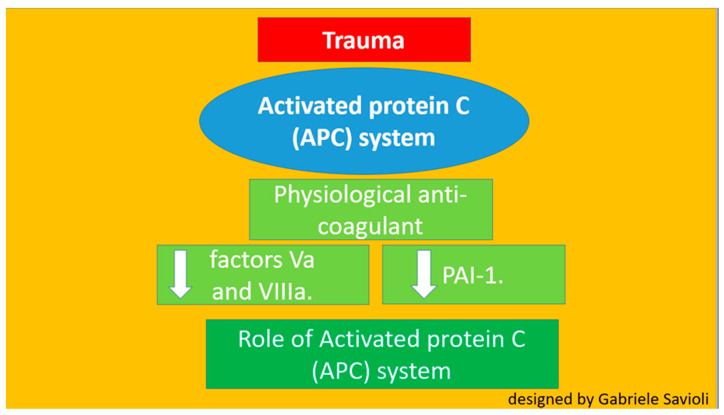
Role of protein C.

Several theories have been postulated regarding the pathophysiological process that triggers TIC [22,23,24,25,26]. Until recently, activated C protein (APC) had been considered one of the main players (Figure 3) [22,23,24]. It was believed that the APC system played the most important role in TIC development. APC is a physiological anticoagulant able to irreversibly inactivate factors Va and VIIIa, which are pro-coagulants. APC also enhances fibrinolysis by inhibiting plasminogen activator inhibitor-1 (PAI-1) and serves a cytoprotective function via anti-apoptotic and anti-inflammatory mechanisms [22,23].

In the PROMMTT study, TIC at arrival to the emergency ward was associated with the depletion of the pro-coagulatory factors I, II, V, VII. VIII, IX, and X and with protein C system activation [24,25,26]. This apparent contradiction is not inexplicable considering the complexity of the response to trauma, encompassing the involvement of several dynamic physiological systems and the release of a multitude of co-interacting mediators.

#### 3.1.2. Role of the Neurohumoral System

Trauma activates the neurohumoral system, leading to increased secretion of inflammatory cytokines and hormones, such as adrenaline and vasopressin. This increased secretion leads to the activation of endothelial cells, resulting in the release of tPA and Weibel–Palade bodies [25]. These factors bind to the endothelium, induce the release of von Willebrand factor, and encourage platelet recruitment [27,28,29,30,31,32,33,34,35,36].

**Figure 4:**
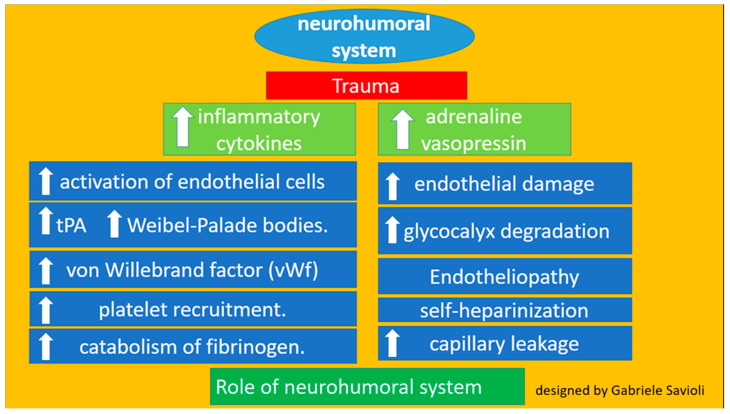
Role of the neurohumoral system.

In addition, the release of tPA and high amounts of plasmin contribute to the catabolism of fibrinogen. This catecholamine increase also damages the endothelium and causes glycocalyx degradation. The process, termed endotheliopathy, may also contribute to capillary leakage following trauma. Specifically, this process induces degradation of the endothelium and the consequent release of glycosaminoglycans such as heparin into circulation and thus activates the phenomenon most properly known as self-heparinization [27,28,29]. Endotheliopathy is present in about 5% of trauma patients and is associated with a high ISS (Figure 4).

Deceased adult patients of trauma have been reported to have presented high levels of adrenaline and syndecan-1. Some studies found high adrenaline levels and glycocalyx damage to be associated with endothelial damage, hyperfibrinolysis, and hypocoagulopathy. Syndecan-1 is an indicator for glycocalyx degradation, and elevated syndecan-1 is associated with an increase in inflammation and endothelial damage [29,30,31,32,33]. Recently both adrenaline and syndecan-1 were proven to be independent predictors of <24-h, 7-day, and 28-day mortality, even after adjustment for ISS [29,30,31,32,33].

Various pathophysiological mechanisms determine whether severe TIC leads to hypofibrinogenemia. In the early phases of trauma TIC induced hypofibrinogenemia is frequently observed. It has been demonstrated that fibrinogen concentrations are less than 2 g/L in approximately 15–20% of patients with TIC, and these low levels were linked to poor outcomes. However, fibrinogen levels may also increase with age [34,35,36].

#### 3.1.3. Role of Platelets

Platelets produce a number of proteins involved in coagulation and fibrinolysis. The mechanisms by which the contradictory activities of secreted platelet proteins affect TIC are unclear. Precise data on platelet function in traumatic patients are scarce: platelet sample handling and specific assays availability are complicating factors in researching the subject [37,38,39].

Studies reported adenosine diphosphate (ADP), arachidonic acid, collagen, and thrombin receptor activating peptide to impair platelet aggregation, suggesting a prevalence of platelet dysfunction of up to 45.5% in patients with trauma on admission and to 91.1% during their stay in the intensive care unit [37,38,39,40,41]. The thrombin receptor pathway has been proposed to play an important role in platelet dysfunction in trauma [42,43,44,45]. However, the mechanisms and implications of these findings are unclear. Anemia, whether caused by hemorrhage or dilution, can also affect platelet adhesion. The available evidence suggests endotheliopathy and anemia to be triggers of platelets dysfunction in trauma.

Some cohorts of massively transfused trauma patients report that measuring platelet count at admission may be used as an outcome predictor, as their platelet count was inversely correlated with injury severity, morbidity, and mortality [37,38,39,40,41,42,43,44,45].

### 3.2. Coagulopathy Associated with Resuscitation Maneuvers

In post-trauma patients, aggressive resuscitation, as previously recommended, with crystalloid dilutes clotting factors and causes metabolic acidosis (hyperchloremic in the case of 0.9% NaCl administration) and interstitial edema. This also caused by microcirculation impairment and impaired oxygen tissue supply [46,47,48]. Colloids cause proteins to move from the blood to the interstitial space, therefore reducing plasma concentration of clotting factors, in particular of factor VII and von Willebrand factor, inhibiting platelet function, and reducing the interaction between factor XIII and fibrin polymers. It has been documented how administering crystalloids in trauma patients worsens TIC, acidemia, and hypothermia, therefore inducing a reduction in thrombin coagulatory activity; it is, therefore, recommended to limit the use of crystalloids in order to reduce coagulation factors dilution effects [48,49,50,51,52,53,54,55,56,57,58,59]. The effects of hypothermia, to which hemorrhage and hypoperfusion contribute, will be discussed more extensively [48,49,51,55,56,57,58].

Finally, acidemia in patients after trauma, which occurs widely, depends on three factors: the use of crystalloids in resuscitation maneuvers, hypoperfusion, and the use of saline solution. In fact, hypoperfusion causes cells to switch from an aerobic mechanism to an anaerobic mechanism, resulting in the production of lactates and a consequent reduction of pH. Saline solution (0.9% NaCl) contains a higher concentration of chlorine than the body under physiological conditions, which could induce hyperchloremic metabolic acidemia [49,50,51,52,53,54].

### 3.3. Detrimental Factors Exacerbating Trauma Coagulopathy

Early trauma induced coagulopathy has been recently recognized as the result of the combination of bleeding-induced shock, tissue injury-related thrombin–thrombomodulin complex generation, and the activation of anticoagulant and fibrinolytic pathways; it is therefore a multifactorial primary condition.

#### 3.3.1. Acidosis

Acidosis is a frequent and early event in patients after trauma that results from inadequate tissue oxygenation, which then activates anaerobic metabolism. Acidosis itself causes plasma protein dysfunction and leads to the rapid degradation of fibrinogen, and almost all stages of clotting are compromised in this setting. At pH less than 7.4, we observe:

- Changes of platelet shapes and structure;
- Reductions of clotting factor activity;
- Compromised thrombin production;
- Reductions of the fibrinogen concentration;
- Increased fibrinogen degradation (caused by increased fibrinolysis and increased factor XIII levels) without effects on fibrinogen production;
- Increased pro-inflammatory responses by platelet-mediated neutrophils;
- Bicarbonate administration to correct acidosis does not correlate with reversal of TIC [49,50,51,52,53,54].

#### 3.3.2. Hypothermia

After trauma heat loss, reduced heat production, and fluid administration can induce hypothermia. Clinically significant reductions of platelet function and coagulation factor activity start at temperatures less than 36 °C and worsen dramatically at temperatures less than 33 °C. Hypothermia influences several key stages of the coagulation process, including the following:

- Negatively affects platelet function;
- Reduces the enzyme activity of clotting factors;
- Induces the activation of fibrinolysis;

The effects are reversible with the normalization of body temperature, which represents a first-level goal to be achieved, both through the use of thermal blankets, by other means of physically warming the patient, or the administration of hot liquids (40 °C).

Overall, the other two components of the lethal triad act on clotting in all phases. In particular, hypothermia extensively inhibits the early stages of the process, whereas acidosis extensively inhibits the propagation and thrombin generation phase. Regarding fibrinogen metabolism, hypothermia inhibits fibrinogen synthesis and acidosis accelerates its degradation. Regarding the response to therapy, we can note some differences in this case. Specifically, the effects of hypothermia are corrected when the body temperature is restored at least 36 °C, whereas the effects of acidosis cannot be immediately corrected with the normalization of pH [48,49,51,55,56,57,58].

#### 3.3.3. Shock

There is agreement that shock is an independent risk factor for TIC even though the true frequency of shock in patients after trauma is unknown. Systolic blood pressure has been used in several studies as the parameter of choice to diagnose hypoperfusion. Traumatic brain Injury (TBI) decreases the precision of using blood pressure as a determinant for hypoperfusion. The systolic blood pressure goals differ depending on the areas involved in MT. In cases of head trauma, systolic pressure must reach 110 mmHg, whereas a value of 90 mmHg is desired when trauma occurs in other regions [22,59,60,61,62,63]. Despite the different kinds of injuries, shock and its effect on the sympathoadrenal system, the endothelium (including the glycocalyx), and hemostatic cells in blood circulation determine the phenotypic features that characterize the clinical conditions of patients with acute critical illness. Catecholamine-induced endothelial damage causes endothelial degradation, which results in glycocalyx shedding, which is the breakdown of tight junctions bringing about capillary leakage, and a pro-coagulant microvasculature that further reduces oxygen delivery because of increased tissue pressure and microvascular thrombosis, creating a vicious circle that ultimately leads to organ failure. Severe trauma, burn injury, and endotoxemia induce similar early genetic responses, indicating that the body response to various acute critical conditions accompanied by shock is relatively homogenous and most likely evolutionarily adapted [64,65,66,67,68,69,70,71,72].

#### 3.3.4. TBI

TIC related to TBI usually occurs within minutes of head injury [18,73] it can be inferred that it is triggered by substances released, following brain damage, at the systemic level through the damaged blood–brain barrier (BBB). BBB is a semipermeable barrier consisting of cells (endothelial, smooth muscle, astrocytes, etc.) and an extracellular matrix [74] responsible for the (active and passive) control of fluids and macromolecules. Head trauma also increases the permeability of the BBB through secondary ischemic and inflammatory lesions. [75,76] Such lesions are mediated by intracellular signals of endothelial cell junction proteins, such as claudins [77,78,79] and junctional adhesion molecules. [80,81] The increase in permeability of the BBB causes fluid leakage with consequent cerebral edema. Cerebral edema in turn contributes to the release of substances involved in the triggering of systemic coagulopathy.

Among these substances it seems that brain-derived cellular microvesicles (BDMV) may play a role both as a diffusion factor and as a causal factor [72,73,82,83,84]. A study has shown in mouse models their rapid release into the circulation associated with a state of systemic hypercoagulability which rapidly evolves into consumption coagulopathy [85].

Their procoagulant power may be due to an abundant expression of the abundant tissue factor and phosphatidylserine [85].

Infusion of purified BDMV resulted in the initiation of a hypercoagulable state in non-trauma mice.

Some studies claim to have detected the fibrinolysis by-product D-Dimer along with other fibrinogen degradation products before detecting an alteration in prothrombin time (PT) and partial thromboplastin time (PTT), which reached their peaks approximately 3–6 h post TBI; some studies suggested these timeframes to be consistent with an early transition from a hypercoagulable to a hypocoagulable state [25,86,87,88,89,90,91]. However, many steps are required to fully understand the role of head trauma in activating TIC, particularly concerning its role in determining changes in fibrinolysis and platelet function. Regarding the changes of fibrinolysis inhibitors, few cases have been reported. Concerning platelet function, patients with TBI appear to have moderately low counts, but often, they are activated, permitting pro-coagulant activity [25,88,89,90,91,92,93,94,95,96,97].

#### 3.3.5. Age, Male Sex and Comorbidities

Coagulopathy is modified by trauma-related factors such as age, sex, and comorbidities including diabetes and hypertension. Significantly different sympathoadrenal and endothelial responses to MT in older and younger patients have been reported. Patient age also appears to significantly influence TIC, including the degree of endotheliopathy. This is congruent to the established correlation between old age and progressive disruption and dysfunction of the endothelium, with the greatest severity of disruption reported in smokers and patients with diabetes, atherosclerosis, or hypertension. Together with age, gender as well has a significant influence over the endogenous trauma-shock response; both age and male sex are independent predictors of multiple-organ failure, a complication closely related to endotheliopathy in major trauma patients [72,98,99,100,101,102,103]. Previously reported comorbidities can lead to worse outcomes, probably also because of endotheliopathy.

#### 3.3.6. Other Factors

In addition to the aforementioned factors, the severity of coagulation disorders is influenced by environmental factors and the resulting therapeutic factors such as the genetic background, inflammation, and premedication, especially with oral anticoagulant use [101,102,103]. Concerning the involvement of anatomical regions, associations were found between TIC and the involvement of the abdominal region [93,101,102,103]. A recent study reported that a higher number of involved regions was correlated with the early development of TIC [93]. Some authors have highlighted the role of some biomarkers, in particular troponin and ultra-sensitive troponin, in highlighting worse outcomes, also due to bleeding, of trauma patients. Their early rise is in fact correlated with worse outcomes and ultra-sensitive troponin could play a role in stratifying even better patients at higher risk. Further studies will be needed to possibly better define a role of these biomarkers in early highlighting coagulopathy related to severe trauma [104,105,106,107].

**Figure 5:**
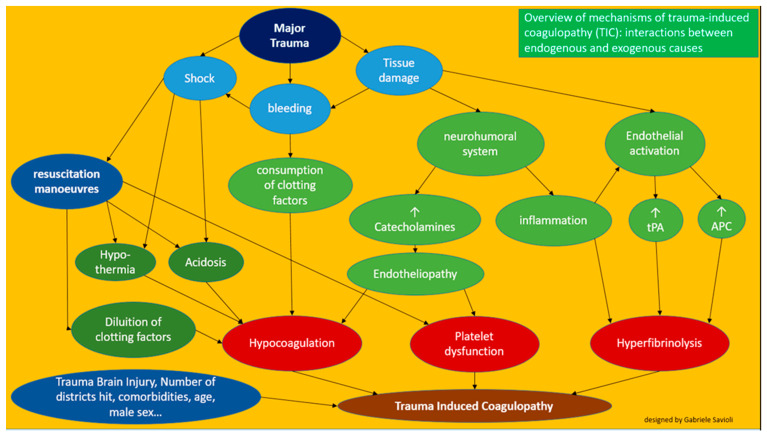
Overview of the mechanisms of trauma-induced coagulopathy (TIC).

Overall the pathophysiological mechanisms of trauma-induced coagulopathy are therefore multi-layered and complex (Figure 5).

## 4. Specials Clinical Forms of TIC

In addition to the aforementioned forms and severity of coagulopathy hyperfibrinolysis, hypocoagulation, then hypercoagulation (hypofibrinolysis)—some other forms are worthy of discussion [108,109,110,111,112,113,114,115,116,117,118,119,120].

### 4.1. Early Primary Hyperfibrinolysis

A limited number of patients experience rapid activation during the early manifestation process of coagulopathy and an uncontrollable pattern of fibrinolysis. This clinical picture is termed early primary hyperfibrinolysis. Hyperfibrinolysis is present in approximately 2.5–7% of all traumatized patients. Early diagnosis of this form presents many difficulties. Viscoelastic tests (see below) highlight only some cases, whereas occult hyperfibrinolysis appears to be more common. This condition may be associated with greater morality, with some authors suggesting rates of 60–80%.

Early administration of anti-fibrinolytics is required as demonstrated by the CRASH two study. The administration of tranexamic acid (TXA) within the first 3 h in patients with active bleeding or those at risk of bleeding is strongly recommended (recommendation class 1A) according to the 2016 and 2019 European guidelines [9,101,121,122].

### 4.2. Late Hypercoagulability

Late coagulopathy has been observed as the hemostatic reaction following trauma, which normalized throughout recovery in uncomplicated patients, whereas patients with severe injuries may experience complications of massive coagulopathy. Recovery from coagulopathy and the return to normal clotting values may be delayed in such patients after severe trauma. A massive physiological response follows trauma, leading to a multitude of changes in the neurohumoral system, the natural pro- and anti-coagulation systems, and other previously reported systems. Distinguishing adaptive from maladaptive systemic inflammatory response to injury remains difficult.

From a clinical point of view, the identification of organ dysfunction could be a reliable indicator of maladaptive systemic inflammation. Multi-organ dysfunction syndrome is present in almost 30% of severely injured patients; it is associated with worse outcomes and a high mortality rate. It is important to remember that late hypercoagulopathy after trauma correlates with an increased risk of venous thromboembolism.

## 5. Diagnosis

### 5.1. Clinical Features

Although blood loss is sometimes noticeable, neither visual evaluation nor physiological parameters are effective guides to understand the degree of hemorrhage. Trauma dynamics is an important tool for identifying patients at risk of significant bleeding. For instance, a threshold of 6 m (20 ft) defines the critical fall height associated with major trauma according to the American College of Surgeons. Additional critical mechanisms include the high-energy deceleration effect and gunshot wounds. The dynamics of trauma combined with severity, the patient’s clinical presentation, and the response to the initial resuscitation maneuvers should further lead to the decision to begin initial hemorrhage control as described in ATLS. An American study by Mutschler et al. analyzed the accuracy of this classification reporting that over 90% of all patients cannot be classified following the ATLS criteria of hypovolemic shock. This system is composed of four classes of patients depending on their vital parameters and state of consciousness [17,121,123,124,125,126].

The same group studied the effectiveness of the ATLS classification criteria and reported that it may underestimate sensory alterations in hypovolemic shock and overestimate the degree of tachycardia associated with hypotension. A three-class scheme with three kinds of response to initial volemic resuscitation was proposed. The first class consists of patients who respond with stable normalization of vital parameters. The second class, comprising transient responders after initial stabilization and volemic filling, subsequently present with unstable vital parameters of consciousness. The third class consists of non-responders to volemic filling. The second and third classes are candidates for immediate surgical management of bleeding [17,124,125,126,127,128].

### 5.2. Laboratory Tests

TIC is diagnosed on the basis of laboratory abnormalities that do not necessarily correspond to distinct clinical phenotypes. Despite coagulation research progress and achievements, an established and verified test to predict and identify clinically relevant acquired coagulopathy is lacking. Current literature on TIC is mostly based on abnormalities of PT, aPTT, plasma fibrinogen concentration, and platelet count, either alone or in combination.

The early identification of coagulopathy in patients with trauma is important, as this can lead to better management and overall improvement in outcomes. The most commonly used tests are traditional clotting tests (aPTT and PT), along with the platelet count and fibrin monitoring. Originally, TIC was defined as an increase in clotting plasma variables such as the aPTT, PT, and international normalized ratio. Emerging evidence suggests that whole-blood viscoelastic tests, such as thromboelastography or rotational thromboelastometry, may better identify coagulopathy and the stage, type, and location of TIC. High ISSs are associated with increases in the severity of TIC and risk of poor outcomes.

Three stages of TIC can be proposed corresponding to more serious clinical frameworks and worse outcomes hyperfibrinolysis, hypocoagulation, then hypercoagulation (hypofibrinolysis). Viscoelastic tests can provide partial results in minutes. They also have the advantage of being able to diagnose, quantify, and classify fibrinolysis, thus allowing the use of anti-fibrinolytic and blood-resistant drugs such as concentrated fibrinogen.

Viscoelastic tests have also been revealed to prevent inappropriate hemotransfusion and hemostatic infusion of blood derivatives to non-coagulopathic patients [17,121,123,124,125,126,127,128]. In addition, the severity of TIC may vary with ongoing treatment, and viscoelastic tests are able to record these changes. Current hematochemical tests (PT, aPTT, fibrinogen, platelets), despite having the advantage of being universally available, require a long time for analysis. In addition, PT and aPTT are only useful for analysis in the early stages of clot formation, and they do not provide a complete view of actual pro-coagulant and anticoagulant activity, in particular on platelets, as well as hyperfibrinolytic activity.

The use instead of viscoelastic tests, such as thromboelastography and thromboelastometry, could remedy these problems, as they more quickly provide a more complete view of the entire clot process, giving a reflected view of the homeostatic process in vivo, including pertinent information regarding the analysis of platelets and fibrinogen, which not provided by routine hematochemical testing [17,121,123,124,125,126,127,128].

## 6. Outcomes

This section synthetically summarizes (because we discussed this topic elsewhere in the text) that patients who develop TIC have worse prognoses regardless of the initial severity. Among the worst complications they face include a higher need for hemotransfusion, a higher rate of hospitalization, a higher rate of hospitalization in intensive care, and a higher mortality rate. We can therefore observe that these patients have worse clinical outcomes and require more hospital and pre-hospital resources. As demonstrated with other pathologies as well, the lack of early recognition and treatment aggravates the outcome [129,130,131,132,133,134].

## 7. Hints for Therapy

Hypovolemic resuscitation, hypothermia prevention, and early clotting support are, together with damage control surgery, the cornerstones of damage control resuscitation (DCR). The convention of DCR largely arose following the discovery of the lethal triad of hypothermia, acidosis, and coagulopathy with the goal of avoiding the initiation of this cycle or reversing its progression. DCR is the strategy by which we attempt to correct the early conditions that promote bleeding and compromise hemostasis and to limit the damage caused by hypoperfusion [135,136,137,138,139,140,141].

International guidelines state that the management of bleeding trauma should follow the principle that the normalization of coagulation parameters improves outcome. It is reasonable to suspect TIC to affect severely injured patients, and therefore a “best guess” treatment should be initiated; during resuscitation a goal-driven approach is considered optimal.

Coagulation support measures should be initiated immediately at admission, and it remains of paramount importance to rapidly assess the type and degree of coagulopathy in the individual patient along with identifying the most prominent causative factors in order to correctly treat the patient in a goal-driven fashion.

Early monitoring of coagulation is essential to detect trauma-induced coagulopathy and define the main causes. Early therapeutic intervention improves coagulation, reduces the need for red blood cell (RBC), fresh frozen plasma (FFP), and platelet transfusion, decreases the incidence of post-traumatic multi-organ failure, shortens the length of hospital stay, and potentially improves survival. The success of early intervention determines the best coagulation management to reduce transfusions and improve outcomes, including reductions of the risk of mortality [8,9,141,142,143,144,145,146,147].

**Figure 6:**
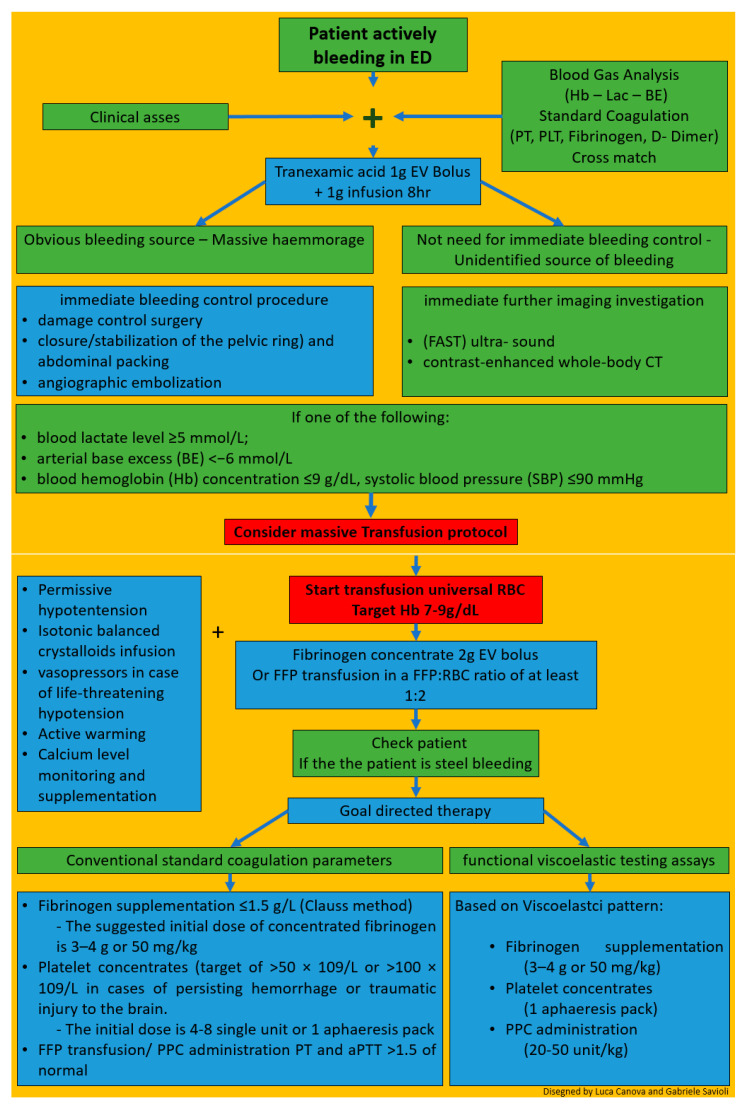
Overview of hints for therapy.

Briefly, we will emphasize some aspects of management for the ED treatment of patients with TIC following the European guideline on the management of major bleeding and coagulopathy following trauma fifth edition published in 2019 (Figure 6) [124].

The first step in the EW is the clinical assessment of the extent of the hemorrhage. a combination of patient physiology, anatomical injury pattern, mechanism of injury and patient response to initial resuscitation can help estimating the severity of the bleeding. (4R,1C).

At the same time, adequate techniques to monitor and promote coagulation should be executed (R23/1B). A blood gas analysis should be performed as soon as possible to obtain hemoglobin (Hb), lactate and base deficit (BE), indicative parameters for shock and the magnitude of hemorrhage with coexisting coagulopathy (R8–9/1B). Blood sample should be collected for standard clotting parameters (prothrombin time, platelet count, and fibrinogen concentration) and/or point-of-care PT/international normalized ratio (INR) (R10/1C) and/or functional viscoelastic testing assays (R10/1C). The 2019 updated European trauma guideline, for the first time, considers standard clotting parameters and viscoelastic testing results as equivalent in the acute assessment of the bleeding trauma patient. Functional assessment of initiation and speed of clot formation, fibrinolytic activity and the functional levels of fibrinogen and platelets can be determined in whole blood by means of viscoelastic tests resulting in accelerate and tailored therapies.

Ongoing this first step, in trauma patients who are bleeding or who are at risk of significant hemorrhage tranexamic acid is to be administered as early as possible at a loading dose of 1 g infused over 10 min, followed by an intravenous (IV) infusion of 1 g over 8 h; administration should be started within 3 h after injury; TXA should not be given more than 3 h after injury (R22/1A).

Immediate bleeding control procedure is recommended in patients with an obvious bleeding source and those with hemorrhagic shock and a suspected source of bleeding (R5/1C) according to the classical damage control procedures (R18/1B) with closure/stabilization of the pelvic ring (R19/1B) and abdominal packing (R20/1B); angiographic embolization may be an option if available. Some clinical studies point out and the European Guidelines recommend performing immediate bleeding control procedure on patients with gunshot wounds and a suspected source of bleeding [122,148,149,150,151].

Immediate further imaging investigation such as: focused assessment with sonography in trauma (FAST) ultra- sound for the detection of free fluid in patients with torso trauma (R7–1C) and contrast-enhanced whole-body CT (WBCT) for the detection and identification of type of injury and potential source of bleeding. (R7–1B) is recommended in patients without a need for immediate bleeding control and an unidentified source of bleeding (R7–1B).

From a practical point of view if one of the following: blood lactate level ≥ 5 mmol/L; arterial base excess (BE) < −6 mmol/L; blood hemoglobin (Hb) concentration ≤ 9 g/dL, systolic blood pressure (SBP) ≤ 90 mmHg is present a predefined massive transfusion protocol MT should be started [152].

Concentrated red blood cells must be transfused to achieve a hemoglobin target of 7–9 g/dL.

Early management of patients with expected massive hemorrhage should follow one of these two strategies [8,9,139,140,141,142,143,144,145,146,147]: the empirical use of fresh frozen plasma (FFP) and packed red blood cell concentrates (pRBC) at a predefined ratio of at least 1:2 (R24/1C) or, alternatively, the use of fibrinogen concentrate and pRBC (R24/1C). Fibrinogen is the substrate for blood to clot and the first coagulation factor which reaches critical thresholds during acute and critical bleeding [17]. Administration of 2 g of fibrinogen to mimic the expected 1:1 ratio corresponding to the first four units of RBC and potentially correct hypofibrinogenemia, if present, has been proposed for initial coagulation support, while waiting for the results of viscoelastic or laboratory tests [152]. Endogenous fibrinogen has been shown experimentally not to be suppressed by fibrinogen administration. Moreover, recent studies have demonstrated a positive trend for survival and saving allogenic blood products when the fibrinogen concentrate approach was followed [153].

Simultaneously to reduce the blood loss, permissive hypotension is recommended with systolic target pressures 80–90 mm Hg (mean target pressure 50–60 mm Hg) in the absence of traumatic brain injury (TBI) until control of bleeding has been achieved (R12/1C). In the presence of TBI, a mean arterial pressure (MAP) ≥ 80 mm Hg is suggested to maintain an adequate cerebral perfusion pressure (R12/1C).

Isotonic balanced crystalloids should be given to achieve the perfusion target (R15/1A), associated with vasopressors in case of life-threatening hypotension and shock (R14/1C).

Heat lost must be avoided and technique to warm the patient should be employed (R17/1C). Calcium levels must be maintained within the reference ranges, especially in settings where a massive transfusion is needed (R30/1C).

After these first steps the patient has to be rechecked and if still bleeding these blind strategies should be replaced by a targeted and tailored strategy, guided either by conventional standard coagulation parameters or by the results from functional viscoelastic testing assays (R25/1B).

If functional viscoelastic testing is not available, the threshold for fibrinogen supplementation is ≤1.5 g/L with the Clauss method (R28/1C). The suggested initial dose of concentrated fibrinogen is 3–4 g or 50 mg/kg. Any repetition must be conducted using laboratory tests. The platelet concentrates should be transfused with a target of >50 × 109/L (R29/1C), or >100 × 109/L in cases of persisting hemorrhage or traumatic injury to the brain (R29/2C).

In this second phase, FFP transfusion should be based on PT and aPTT (>1.5 of normal) value and/or viscoelastic patterns (R26/1C). The administration of FFP in the absence of massive bleeding (R26/1B) or to correct hypofibrinogenemia is not advised (R26/1C).

Coagulation factor concentrates (PPC) has proven better than FFP in rapidly reversing vitamin K antagonists, as there is evidence of decreased hematoma formation in head trauma patients. PCC is therefore the preferred choice for vitamin k antagonists effect reversal. PCC are, for the first time, allowed by European updated Guidelines in case of lack of coagulation factors diagnosed by standard coagulation or, better, viscoelastic patterns (R26/1C).

The identification and management of patients pre-treated with anticoagulant agents, especially direct anticoagulant, continues to pose a major challenge despite accumulating experience and awareness [12,14,15,16,17,44,59,74,101,102,103,122,154,155,156,157,158,159]. Idarucizumab is indicated as an antidote for the thrombin inhibitor dabigatran (5 g intravenously (R35/1B). In case of severe and life-threatening hemorrhage under preinjury factor-Xa inhibition, the recommended treatment is combined TXA 15 mg/kg (or 1 g) and PCC (25–50 units/kg) (R34/2C). The European Medicines Agency (EMA) supported the approval of factor-Xa antidote andexanet alfa and the agent became available in most European countries [160].

Platelet concentrates administration is recommended in case of documented platelet dysfunction and/or in patients with persistent bleeding previously treated with platelet inhibitors (R36/2C); this is to be considered in particular in patients with intracranial hemorrhage in need for an acute neurosurgical intervention (R36/2B) with the possible additional use of desmopressin (R36/2C).

## 8. Management of Patients with Severe Trauma in the ED

Briefly, we will emphasize some aspects of management for the ED treatment of patients with TIC.

The findings of this review highlighted the need for protocols for the management of coagulopathy regarding diagnostics and therapeutic pathways in patients with severe trauma in line with the most up-to-date guidelines. The usefulness of protocols for massive hemotransfusion and the need for bedside clotting analyzers have also been demonstrated. The European guidelines currently recommend to directly transfer patients to an appropriate trauma center for treatment and to follow a restricted volume replacement strategy during initial resuscitation [1,2,3,5,6,7,8,9,10,11,101,121,122,151,154,161,162,163,164,165,166,167,168,169,170]. Blood product optimal use procedures continue to evolve, and their development should be goal directed. Despite greater awareness and experience, the identification and management of patients under the effects of anti-coagulant agents remains a major challenge [12,14,15,16,17,59,74,101,102,103,122,154,155,156,157,158,159,162].

## 9. Conclusions

- TIC is a dynamic sequence coagulation disorder from hyperfibrinolysis, hypercoagulation to its final stage hypocoagulation. The early hypocoagulable state is not related to dilution nor to iatrogenic or hypothermic causes.
- TIC is present in approximately one-third of patients who report MT
- The physiopathology of TIC is complex and features several contributing causes. The role of protein C has been less emphasized
- The diagnosis and management of TIC often encompasses standard coagulation test and functional viscoelastic assays.
- Early initiation of antifibrinolytic therapy and balanced resuscitation of coagulation disorder is the mainstay of TIC
- TIC is related to worse outcomes, among which increased rates of transfusion, infection, thromboembolism, acute lung injury, multi-organ failure, and death.

## Figures and Tables

**Table 1:** Triage criteria for severe trauma.

Physiological Criteria Anatomical Criteria Dynamic Criteria
Ejection from a vehicle Penetrating head/neck/throat/abdomen/pelvic/armpit/groin trauma Systolic blood pressure < 90 mmHg
Motorcycle crash with separation of the rider Amputations above the wrist or ankle Respiratory distress or respiratory rate of <10 or >29 breaths/min
Died in the same vehicle Chest trauma with flap/flail chest State of consciousness (GCS < 13)
CRASH intrusion >30 cm at the patient area Neurological injury with paralysis of even a single limb
Fall from height >2 m Fractures of two or more long bones
Pedestrian thrown or run over or hit at a speed >10 km/h Suspected unstable fracture ring of pelvis: Suspected unstable fracture
High-energy impact
(Speed > 65 km/h) Open or depressed skull fracture
Vehicle crash Burn >20% of the body surface or airway/face
Extrication time > 20 min

The criteria for activating the severe trauma protocol in our trauma center are presented, including physiological, anatomic, and dynamic criteria for defining probable severe trauma (one of the following criteria is sufficient). GCS, Glasgow coma score.

## Data Availability

Not applicable.

## References

[1] Department of Violence and Injury Prevention and Disability, World Health Organization Injuries and Violence: The Facts. http://whqlibdoc.who.int/publications/2010/9789241599375_eng.pdf.

[2] GBD 2013 Mortality and Causes of Death Collaborators (2014). Global, regional, and national age–sex specific all-cause and cause-specific mortality for 240 causes of death, 1990–2013: A systematic analysis for the Global Burden of Disease Study 2013. Lancet.

[3] Soreide K. (2009). Epidemiology of major trauma. Br. J. Surg..

[4] Frith D., Goslings J.C., Gaarder C., Maegele M., Cohen M.J., Allard S., Johansson P.I., Stanworth S., Thiemermann C., Brohi K. (2010). Definition and drivers of acute traumatic coagulopathy: Clinical and experimental investigations. J. Thromb. Haemost..

[5] Maegele M., Lefering R., Yucel N., Tjardes T., Rixen D., Paffrath T., Simanski C., Neugebauer E., Bouillon B. (2007). Early coagulopathy in multiple injury: An analysis from the German Trauma Registry on 8724 patients. Injury.

[6] Brohi K., Singh J., Heron M., Coats T. (2003). Acute traumatic coagulopathy. J. Trauma.

[7] MacLeod J.B., Lynn M., McKenney M.G., Cohn S.M., Murtha M. (2003). Early coagulopathy predicts mortality in trauma. J. Trauma.

[8] Schöchl H., Nienaber U., Maegele M., Hochleitner G., Primavesi F., Steitz B., Arndt C., Hanke A., Voelckel W., Solomon C. (2011). Transfusion in trauma: Thromboelastometry-guided coagulation factor concentrate-based therapy versus standard fresh frozen plasma-based therapy. Crit. Care.

[9] Schöchl H., Frietsch T., Pavelka M., Jambor C. (2009). Hyperfibrinolysis after major trauma: Differential diagnosis of lysis patterns and prognostic value of thrombelastometry. J. Trauma.

[10] Maegele M., Schochl H., Cohen M.J. (2014). An update on the coagulopathy of trauma. Shock.

[11] Khan S., Davenport R., Raza I., Glasgow S., De’Ath H.D., Johansson P.I., Curry N., Stanworth S., Gaarder C., Brohi K. (2015). Damage control resuscitation using blood component therapy in standard doses has a limited effect on coagulopathy during trauma hemorrhage. Intensive Care Med..

[12] Hagemo J.S., Christiaans S.C., Stanworth S.J., Brohi K., Johansson P.I., Goslings J.C., Naess P.A., Gaarder C. (2015). Detection of acute traumatic coagulopathy and massive transfusion requirements by means of rotational thromboelastometry: An international prospective validation study. Crit. Care.

[13] Savioli G., Ceresa I.F., Macedonio S., Gerosa S., Belliato M., Iotti G.A., Luzzi S., Del Maestro M., Mezzini G., Giotta Lucifero A. (2020). Trauma Coagulopathy and Its Outcomes. Medicina.

[14] Hagemo J.S., Stanworth S., Juffermans N.P., Brohi K., Cohen M., Johansson P.I., Roislien J., Eken T., Naess P.A., Gaarder C. (2014). Prevalence, predictors and outcome of hypofibrinogenaemia in trauma: A multicentre observational study. Crit. Care.

[15] Hess J.R., Brohi K., Dutton R.P., Hauser C.J., Holcomb J.B., Kluger Y., Mackway-Jones K., Parr M.J., Rizoli S.B., Yukioka T. (2008). The coagulopathy of trauma: A review of mechanisms. J. Trauma.

[16] Frith D., Davenport R., Brohi K. (2012). Acute traumatic coagulopathy. Curr. Opin. Anaesthesiol..

[17] Spivey M., Parr M.J. (2005). Therapeutic approaches in trauma-induced coagulopathy. Minerva Anestesiol..

[18] Engels P.T., Rezende-Neto J.B., Al Mahroos M., Scarpelini S., Rizoli S.B., Tien H.C. (2011). The natural history of trauma-related coagulopathy: Implications for treatment. J. Trauma.

[19] Hoffman M., Monroe D.M.I.I.I. (2001). A cell-based model of hemostasis. Thromb. Haemost..

[20] Kushimoto S., Kudo D., Kawazoe Y. (2017). Acute traumatic coagulopathy and trauma-induced coagulopathy: An overview. J. Intensive Care.

[21] Levi M., van der Poll T. (2008). The role of natural anticoagulants in the pathogenesis and management of systemic activation of coagulation and inflammation in critically ill patients. Semin. Thromb. Hemost..

[22] Cohen M.J., Kutcher M., Redick B., Nelson M., Call M., Knudson M.M., Schreiber M.A., Bulger E.M., Muskat P., Alarcon L.H. (2013). Clinical and mechanistic drivers of acute traumatic coagulopathy. J. Trauma Acute Care Surg..

[23] Chesebro B.B., Rahn P., Carles M., Esmon C.T., Xu J., Brohi K., Frith D., Pittet J.F., Cohen M.J. (2009). Increase inactivated protein C mediates acute traumatic coagulopathy in mice. Shock.

[24] Cohen M.J., Call M., Nelson M., Calfee C.S., Esmon C.T., Brohi K., Pittet J.F. (2012). Critical role of activated protein C in early coagulopathy and later organ failure, infection and death in trauma patients. Ann. Surg..

[25] Chapman M.P., Moore E.E., Moore H.B., Gonzalez E., Gamboni F., Chandler J.G., Mitra S., Ghasabyan A., Chin T.L., Sauaia A. (2016). Overwhelming tPA release, not PAI-1 degradation, is responsible for hyperfibrinolysis in severely injured trauma patients. J. Trauma Acute Care Surg..

[26] Gando S., Mayumi T., Ukai T. (2018). Activated protein C plays nomajor roles in the inhibition of coagulation or increased fibrinolysis in acute coagulopathy of trauma-shock: A systematic review. Thromb. J..

[27] Johansson P.I., Stensballe J., Rasmussen L.S., Ostrowski S.R. (2012). High circulating adrenaline levels at admission predict increased mortality after trauma. J. Trauma Acute Care Surg..

[28] Ostrowski S.R., Henriksen H.H., Stensballe J., Gybel-Brask M., Cardenas J.C., Baer L.A., Cotton B.A., Holcomb J.B., Wade C.E., Johansson P.I. (2017). Sympathoadrenal activation and endotheliopathy are drivers of hypocoagulability and hyperfibrinolysis in trauma: A prospective observational study of 404 severely injured patients. J. Trauma Acute Care Surg..

[29] Johansson P.I., Stensballe J., Rasmussen L.S., Ostrowski S.R. (2011). A high admission syndecan-1 level, a marker of endothelial glycocalyx degradation, is associated with inflammation, protein C depletion, fibrinolysis, and increased mortality in trauma patients. Ann. Surg..

[30] Ostrowski S.R., Johansson P.I. (2012). Endothelial glycocalyx degradation induces endogenous heparinization in patients with severe injury and early traumatic coagulopathy. J. Trauma Acute Care Surg..

[31] Rahbar E., Cardenas J.C., Baimukanova G., Usadi B., Bruhn R., Pati S., Ostrowski S.R., Johansson P.I., Holcomb J.B., Wade C.E. (2015). Endothelial glycocalyx shedding and vascular permeability in severely injured trauma patients. J. Transl. Med..

[32] Johansson P.I., Henriksen H.H., Stensballe J., Gybel-Brask M., Cardenas J.C., Baer L.A., Cotton B.A., Holcomb J.B., Wade C.E., Ostrowski S.R. (2017). Traumatic endotheliopathy: A prospective observational study of 424 severely injured patients. Ann. Surg..

[33] Xu L., Yu W.K., Lin Z.L., Tan S.J., Bai X.W., Ding K., Li N. (2015). Chemical sympathectomy attenuates inflammation, glycocalyx shedding and coagulation disorders in ratswithacutetraumaticcoagulopathy. Blood Coagul. Fibrinolysis.

[34] Paydar S., Dalfardi B., Shayan Z., Shayan L., Saem J., Bolandparvaz S. (2018). Early Predictive Factors of Hypofibrinogenemia in Acute Trauma Patients. J. Emerg. Trauma Shock..

[35] McQuilten Z.K., Wood E.M., Bailey M., Cameron P.A., Cooper D.J. (2017). Fibrinogen is an independent predictor of mortality in major traumapatients:a five-year statewidecohortstudy. Injury.

[36] Ohmori T., Kitamura T., Tanaka K., Saisaka Y., Ishihara J., Onishi H., Nojima T., Yamamoto K., Matusmoto T., Tokioka T. (2015). Admission fibrinogen levels in severe trauma patients: A comparison of elderly and younger patients. Injury.

[37] Wohlauer M.V., Moore E.E., Thomas S., Sauaia A., Evans E., Harr J., Silliman C.C., Ploplis V., Castellino F.J., Walsh M. (2012). Early platelet dysfunction: An unrecognized role in the acute coagulopathy of trauma. J. Am. Coll. Surg..

[38] Kutcher M.E., Redick B.J., McCreery R.C., Crane I.M., Greenberg M.D., Cachola L.M., Nelson M.F., Cohen M.J. (2012). Characterization of plateletdysfunctionaftertrauma. J. Trauma Acute Care Surg..

[39] Ramsey M.T., Fabian T.C., Shahan C.P., Sharpe J.P., Mabry S.E., Weinberg J.A., Croce M.A., Jennings L.K. (2016). A prospective study of platelet function in trauma patients. J. Trauma Acute Care Surg..

[40] Sirajuddin S., Valdez C., DePalma L., Maluso P.J., Singhal R., Schroeder M., Sarani B. (2016). Inhibition of platelet function is common following even minor injury. J. Trauma Acute Care Surg..

[41] Schnuriger B., Inaba K., Abdelsayed G.A., Lustenberger T., Eberle B.M., Barmparas G., Talving P., Demetriades D. (2010). The impact of platelets on the progression of traumatic intracranial hemorrhage. J. Trauma.

[42] Hess J.R., Lindell A.L., Stansbury L.G., Dutton R.P., Scalea T.M. (2009). The prevalence of abnormal results of conventional coagulation tests on admission to a trauma center. Transfusion.

[43] Brown L.M., Call M.S., Knudson M.M., Cohen M.J., Trauma Outcomes Group (2011). A normal platelet count may not be enough: The impact of admission platelet count on mortality and transfusion in severely injured trauma patients. J. Trauma.

[44] Floccard B., Rugeri L., Faure A., Saint Denis M., Boyle E.M., Peguet O., Levrat A., Guillaume C., Marcotte G., Vulliez A. (2012). Early coagulopathy in trauma patients: An on-scene and hospital admission study. Injury.

[45] Van Beek J.G., Mushkudiani N.A., Steyerberg E.W., Butcher I., McHugh G.S., Lu J., Marmarou A., Murray G.D., Maas A.I.R. (2007). Prognostic value of admission laboratory parameters in traumatic brain injury: Results from the IMPACT study. J. Neurotrauma..

[46] Szentkereszty Z. (2020). Az akut traumás vérzés és véralvadási zavar korszerű ellátása [Up-to-date management of acute traumatic bleeding and coagulopathy]. Orv. Hetil..

[47] Cole E., Weaver A., Gall L., West A., Nevin D., Tallach R., O’Neill B., Lahiri S., Allard S., Tai N. (2019). A decade of damage control resuscitation: New transfusion practice, new survivors, new directions. Ann. Surg..

[48] Mitrophanov A.Y., Rosendaal F.R., Reifman J. (2013). Computational analysis of the effects of reduced temperature on thrombin generation: The contributions of hypothermia to coagulopathy. Anesth. Analg..

[49] Meng Z.H., Wolberg A.S., Monroe D.M.I.I.I., Hoffman M. (2003). The effect of temperature and pH on the activity of factor VIIa: Implications forthe efficacy of high-dose factor VIIa in hypothermic and acidotic patients. J. Trauma.

[50] Engström M., Schött U., Romner B., Reinstrup P. (2006). Acidosis impairs the coagulation: A thromboelastographic study. J. Trauma.

[51] Martini W.Z., Pusateri A.E., Uscilowicz J.M., Delgado A.V., Holcomb J.B. (2005). Independent contributions of hypothermia and acidosis to coagulopathyin swine. J. Trauma.

[52] Martini W.Z., Holcomb J.B. (2007). Acidosis and coagulopathy: The differential effects on fibrinogen synthesis and breakdown in pigs. Ann. Surg..

[53] Martini W.Z., Dubick M.A., Pusateri A.E., Park M.S., Ryan K.L., Holcomb J.B. (2006). Does bicarbonate correct coagulation function impaired by acidosis in swine?. J. Trauma.

[54] Shenkman B., Budnik I., Einav Y., Hauschner H., Andrejchin M., Martinowitz U. (2017). Model of trauma-induced coagulopathy including hemodilution, fibrinolysis, acidosis, and hypothermia: Impact on blood coagulation and platelet function. J. Trauma Acute Care Surg..

[55] Wolberg A.S., Meng Z.H., Monroe D.M.I.I.I., Hoffman M. (2004). A systematic evaluation of the effect of temperature on coagulation enzyme activity and platelet function. J. Trauma.

[56] Mitrophanov A.Y., Szlam F., Sniecinski R.M., Levy J.H., Reifman J. (2020). Controlled Multifactorial Coagulopathy: Effects of Dilution, Hypothermia, and Acidosis on Thrombin Generation in Vitro. Anesth Analg..

[57] Martini W.Z. (2009). Coagulopathy by hypothermia and acidosis: Mechanisms of thrombin generation and fibrinogen availability. J. Trauma.

[58] De Robertis E., Kozek-Langenecker S.A., Tufano R., Romano G.M., Piazza O., Zito Marinosci G. (2015). Coagulopathy induced by acidosis, hypothermia and hypocalcaemia in severe bleeding. Minerva Anestesiol..

[59] Brohi K., Cohen M.J., Ganter M.T., Schultz M.J., Levi M., Mackersie R.C., Pittet J.F. (2008). Acute coagulopathy of trauma: Hypoperfusion induces systemic anticoagulation and hyperfibrinolysis. J. Trauma.

[60] Jansen J.O., Scarpelini S., Pinto R., Tien H.C., Callum J., Rizoli S.B. (2011). Hypoperfusion in severely injured trauma patients is associated with reduced coagulation factor activity. J. Trauma.

[61] Lechleuthner A., Lefering R., Bouillon B., Lentke E., Vorweg M., Tiling T. (1994). Prehospital detection of uncontrolled haemorrhage in blunt trauma. Eur. J. Emerg. Med..

[62] Gando S., Sawamura A., Hayakawa M. (2011). Trauma, Shock and disseminated intravascular coagulation: Lessons from the classical literature. Ann. Surg..

[63] Adrie C., Laurent I., Monchi M., Cariou A., Dhainaou J.F., Spaulding C. (2004). Post resuscitation disease after cardiac arrest: A sepsis-like syndrome?. Curr. Opin. Crit. Care.

[64] Johansson P.I., Ostrowski S.R. (2010). Acute coagulopathy of trauma: Balancing progressive catecholamine induced endothelial activation and damage by fluid phase anticoagulation. Med. Hypotheses.

[65] Neumar R.W., Nolan J.P., Adrie C., Aibiki M., Berg R.A., Bottiger B.W., Callaway C., Clark R.S., Geocadin R.G., Jauch E.C. (2008). Post-cardiac arrest syndrome: Epidemiology, pathophysiology, treatment, and prognostication. A consensus statement from the International Liaison Committee on Resuscitation (American Heart Association, Australian and New Zealand Council on Resuscitation, European Resuscitation Council, Heart and Stroke Foundation of Canada, InterAmerican Heart Foundation, Resuscitation Council of Asia, and the Resuscitation Council of Southern Africa); the American Heart Association Emergency Cardiovascular Care Committee; the Council on Cardiovascular Surgery and Anesthesia; the Council on Cardiopulmonary, Perioperative, and Critical Care; the Council on Clinical Cardiology; and the Stroke Council. Circulation.

[66] Opal S.M., van der Poll T. (2015). Endothelial barrier dysfunction in septic shock. J. Intern. Med..

[67] Holcomb J.B. (2011). A novel and potentially unifying mechanism for shock induced early coagulopathy. Ann. Surg..

[68] Cohen J., Vincent J.L., Adhikari N.K., Machado F.R., Angus D.C., Calandra T., Jaton K., Giulieri S., Delaloye J., Opal S. (2015). Sepsis: A roadmap for future research. Lancet Infect Dis..

[69] Adrie C., Adib-Conquy M., Laurent I., Monchi M., Vinsonneau C., Fitting C., Fraisse F., Dinh-Xuan A.T., Carli P., Spaulding C. (2002). Successful cardiopulmonary resuscitation after cardiac arrest as a “sepsis-like” syndrome. Circulation.

[70] Xiao W., Mindrinos M.N., Seok J., Cuschieri J., Cuenca A.G., Gao H., Hayden D.L., Hennessy L., Moore E.E., Minei J.P. (2011). A genomic storm in critically injured humans. J. Exp. Med..

[71] Zhang J., Zhang F., Dong J.F. (2018). Coagulopathy induced by traumatic brain injury: Systemic manifestation of a localized injury. Blood.

[72] Johansson P., Stensballe J., Ostrowski S. (2017). Shock induced endotheliopathy (SHINE) in acute critical illness—A unifying pathophysiologic mechanism. Crit. Care.

[73] Stein S.C., Smith D.H. (2004). Coagulopathy in traumatic brain injury. Neurocrit. Care.

[74] Mitra B., Cameron P.A., Mori A., Fitzgerald M. (2012). Acute coagulopathy and early deaths post major trauma. Injury.

[75] Baskett P.J. (1999). Recommendations for uniform reporting of data following major trauma--the Utstein style. A report of a working party of the International Trauma Anaesthesia and Critical Care Society (ITACCS). Resuscitation.

[76] Komarova Y.A., Kruse K., Mehta D., Malik A.B. (2017). Protein Interactions at Endothelial Junctions and Signaling Mechanisms Regulating Endothelial Permeability. Circ. Res..

[77] Horng S., Therattil A., Moyon S., Gordon A., Kim K., Argaw A.T., Hara Y., Mariani J.N., Sawai S., Flodby P. (2017). Astrocytic tight junctions control inflammatory CNS lesion pathogenesis. J. Clin. Investig..

[78] Cristante E., McArthur S., Mauro C., Maggioli E., Romero I.A., Wylezinska-Arridge M., Couraud P.O., Lopez-Tremoleda J., Christian H.C., Weksler B.B. (2013). Identification of an essential endogenous regulator of blood-brain barrier integrity, and its pathological and therapeutic implications. Proc. Natl. Acad. Sci. USA.

[79] Haseloff R.F., Dithmer S., Winkler L., Wolburg H., Blasig I.E. (2015). Transmembrane proteins of the tight junctions at the blood-brain barrier: Structural and functional aspects. Semin Cell Dev. Biol..

[80] Wójciak-Stothard B., Potempa S., Eichholtz T., Ridley A.J. (2001). Rho and Rac but not Cdc42 regulate endothelial cell permeability. J. Cell Sci..

[81] Tsukita S., Furuse M. (2000). The structure and function of claudins, cell adhesion molecules at tight junctions. Ann. N Y Acad. Sci..

[82] Maegele M. (2013). Coagulopathy after traumatic brain injury: Incidence, pathogenesis, and treatment options. Transfusion.

[83] Lustenberger T., Talving P., Kobayashi L., Inaba K., Lam L., Plurad D., Demetriades D. (2010). Time course of coagulopathy in isolated severe traumatic brain injury. Injury.

[84] Nakae R., Takayama Y., Kuwamoto K., Naoe Y., Sato H., Yokota H. (2016). Time course of coagulation and fibrinolytic parameters in patients with traumatic brain injury. J. Neurotrauma.

[85] Aurrand-Lions M., Johnson-Leger C., Wong C., Du Pasquier L., Imhof B.A. (2001). Heterogeneity of endothelial junctions is reflected by differential expression and specific subcellular localization of the three JAM family members. Blood.

[86] Fleck R.A., Rao L.V., Rapaport S.I., Varki N. (1990). Localization of human tissue factor antigen by immunostaining with monospecific, polyclonal anti-human tissue factor antibody. Thromb. Res..

[87] Eddleston M., de la Torre J.C., Oldstone M.B., Loskutoff D.J., Edgington T.S., Mackman N. (1993). Astrocytes are the primary source of tissue factor in the murine central nervous system. A role for astrocytes in cerebral hemostasis. J Clin Investig..

[88] Karri J., Cardenas J.C., Matijevic N., Wang Y.W., Choi S., Zhu L., Cotton B.A., Kitagawa R., Holcomb J.B., Wade C.E. (2017). Early fibrinolysis associated with hemorrhagic progression following traumatic brain injury. Shock.

[89] Hijazi N., Abu Fanne R., Abramovitch R., Yarovoi S., Higazi M., Abdeen S., Basheer M., Maraga E., Cines D.B., Higazi A.A.R. (2015). Endogenous plasminogen activators mediate progressive intracerebral hemorrhage after traumatic brain injury in mice. Blood.

[90] Wu X., Darlington D.N., Cap A.P. (2016). Procoagulant and fibrinolytic activity after polytrauma in rat. Am. J. Physiol. Regul. Integr. Comp. Physiol..

[91] Ploplis V.A., Donahue D.L., Sandoval-Cooper M.J., MorenoCaffaro M., Sheets P., Thomas S.G., Walsh M., Castellino F.J. (2014). Systemic platelet dysfunction is the result of local dysregulated coagulation and platelet activation in the brain in a rat model of isolated traumatic brain injury. J. Neurotrauma.

[92] Prodan C.I., Vincent A.S., Dale G.L. (2016). Coated-platelet levels increase with number of injuries in patients with mild traumatic brain injury. J. Neurotrauma.

[93] Savioli G., Ceresa I.F., Ciceri L., Sciutti F., Belliato M., Iotti G.A., Luzzi S., Del Maestro M., Mezzini G., Lafe E. (2020). Mild head trauma in elderly patients: Experience of an emergency department. Heliyon.

[94] Morel N., Morel O., Petit L., Hugel B., Cochard J.F., Freyssinet J.M., Sztark F., Dabadie P. (2008). Generation of procoagulant microparticles in cerebrospinal fluid and peripheral blood after traumatic brain injury. J. Trauma.

[95] Broekhuizen L.N., Mooij H.L., Kastelein J.J., Stroes E.S., Vink H., Nieuwdorp M. (2009). Endothelial glycocalyx as potential diagnostic and therapeutic target in cardiovascular disease. Curr. Opin. Lipidol..

[96] Johansson P.I., Sørensen A.M., Perner A., Welling K.L., Wanscher M., Larsen C.F., Ostrowski S.R. (2012). Elderly trauma patients have high circulating noradrenaline levels but attenuated release of adrenaline, platelets and leukocytes in response to increasing injury severity. Crit. Care Med..

[97] Ostrowski S.R., Pedersen S.H., Jensen J.S., Mogelvang R., Johansson P.I. (2013). Acute myocardial infarction is associated with endothelial glycocalyx and cell damage and a parallel increase in circulating catecholamines. Crit. Care.

[98] Schreiber M.A., Differding J., Thorborg P., Mayberry J.C., Mullins R.J. (2005). Hypercoagulability is most prevalent early after injury and in female patients. J. Trauma.

[99] Frohlich M., Lefering R., Probst C., Paffrath T., Schneider M.M., Maegele M., Sakka S.G., Bouillon B., Wafaisade A. (2014). Epidemiology and risk factors of multipleorgan failure after multiple trauma: An analysis of 31,154 patients from the TraumaRegister DGU. J. Trauma Acute Care Surg..

[100] Savioli G., Ceresa I.F., Luzzi S., Gragnaniello C., Giotta Lucifero A., Del Maestro M., Marasco S., Manzoni F., Ciceri L., Gelfi E. (2020). Rates of Intracranial Hemorrhage in Mild Head Trauma Patients Presenting to Emergency Department and Their Management: A Comparison of Direct Oral Anticoagulant Drugs with Vitamin K Antagonists. Medicina.

[101] Hess J.R., Lawson J.H. (2006). The coagulopathy of trauma versus disseminated intravascular coagulation. J. Trauma.

[102] Spahn D.R., Rossaint R. (2005). Coagulopathy and blood component transfusion in trauma. Br. J. Anaesth..

[103] Hussmann B., Lefering R., Waydhas C., Touma A., Kauther M.D., Ruchholtz S., Lendemans S. (2013). Does increased prehospital replacement volume lead to a poor clinical course and an increased mortality? A matched-pair analysis of 1896 patients of the Trauma Registry of the German Society for Trauma Surgery who were managed by an emergency doctor at the accident site. Injury.

[104] Palmeri D., van Zante A., Huang C.C., Hemmerich S., Rosen S.D. (2000). Vascular endothelial junction-associated molecule, a novel member of the immunoglobulin superfamily, is localized to intercellular boundaries of endothelial cells. J. Biol. Chem..

[105] Tian Y., Salsbery B., Wang M., Yuan H., Yang J., Zhao Z., Wu X., Zhang Y., Konkle B.A., Thiagarajan P. (2015). Brain-derived microparticles induce systemic coagulation in a murine model of traumatic brain injury. Blood.

[106] Keskpaik T., Starkopf J., Kirsimägi Ü., Mihnovitš V., Lomp A., Raamat E.M., Saar S., Talving P. (2020). The role of elevated high-sensitivity cardiac troponin on outcomes following severe blunt chest trauma. Injury.

[107] Kalbitz M., Pressmar J., Stecher J., Weber B., Weiss M., Schwarz S., Miltner E., Gebhard F., Huber-Lang M. (2017). The Role of Troponin in Blunt Cardiac Injury After Multiple Trauma in Humans. World J Surg..

[108] McCully B.H., Connelly C.R., Fair K.A., Holcomb J.B., Fox E.E., Wade C.E., Bulger E.M., Schreiber M.A., del Junco D.J., Matijevic N. (2017). Onset of coagulation function recovery is delayed in severely injured trauma patients with venous thromboembolism. J. Am. Coll. Surg..

[109] Tompkins R.G. (2015). Genomics of injury: The glue grant experience. J. Trauma Acute Care Surg..

[110] Lord J.M., Midwinter M.J., Chen Y.F., Belli A., Brohi K., Kovacs E.J., Konderman L., Kubes P., Lilford R.J. (2014). The systemic immune response to trauma: An overview of pathophysiology and treatment. Lancet.

[111] Bortolotti P., Faure E., Kipnis E. (2018). Inflammasomes in tissue damages and immune disorders after trauma. Front. Immunol..

[112] Singer M., Deutschman C.S., Seymour C.W., Shankar-Hari M., Annane D., Bauer M., Bellomo R., Bernard G.R., Chiche J.D., Coopersmith C.M. (2016). The Third International Consensus Definitions for Sepsis and Septic Shock (Sepsis-3). JAMA.

[113] Minei J.P., Cuschieri J., Sperry J., Moore E.E., West M.A., Harbrecht B.G., O’Keefe G.E., Cohen M.J., Moldawer L.L., Tompkins R.G. (2012). The changing pattern and implications of multiple organ failure after blunt injury with hemorrhagic shock. Crit. Care Med..

[114] Levi M., van der Poll T. (2017). Coagulation and sepsis. Thromb. Res..

[115] Dhainaut J.F., Yan S.B., Joyce D.E., Pettilä V., Basson B., Brandt J.T., Sundin D.P., Levi M. (2004). Treatment effects of drotrecogin alfa (activated) in patients with severe sepsis with or without overt disseminated intravascular coagulation. J. Thromb. Haemost..

[116] Van Haren R.M., Valle E.J., Thorson C.M., Jouria J.M., Busko A.M., Guarch G.A., Namias N., Livingstone A.S., Proctor K.G. (2014). Hypercoagulability and other risk factors in traumaintensive care unit patients with venous thromboembolism. J. Trauma Acute Care Surg..

[117] Hamada S.R., Espina C., Guedj T., Buaron R., Harrois A., Figueiredo S., Duranteau J. (2017). High level of venous thromboembolism in critically ill trauma patients despite early and well-driven thromboprophylaxis protocol. Ann. Intensive Care.

[118] Skrifvars M.B., Bailey M., Presneill J., French C., Nichol A., Little L., Duranteau J., Huet O., Haddad S., Arabi Y. (2017). Venous thromboembolic events in critically ill traumatic brain injury patients. Intensive Care Med..

[119] Van Gent J.M., Calvo R.Y., Zander A.L., Olson E.J., Sise C.B., Sise M.J., Shackford S.R. (2017). Risk factors for deep vein thrombosis and pulmonary embolism after traumatic injury: A competing risks analysis. J. Trauma Acute Care Surg..

[120] Sumislawski J.J., Kornblith L.Z., Conroy A.S., Callcut R.A., Cohen M.J. (2018). Dynamic coagulability after injury: Is delaying venous thromboembolism chemoprophylaxis worth the wait?. J. Trauma Acute Care Surg..

[121] Mutschler M., Nienaber U., Brockamp T., Wafaisade A., Wyen H., Peiniger S., Paffrath T., Bouillon B., Maegele M. (2013). TraumaRegister DGU A critical reappraisal of the ATLS classification of hypovolaemic shock: Does it really reflect clinical reality?. Resuscitation.

[122] Rugeri L., Levrat A., David J.S., Delecroix E., Floccard B., Gros A., Allaouchiche B., Negrier C. (2007). Diagnosis of early coagulation abnormalities in trauma patients by rotation thrombelastography. J. Thromb. Haemost..

[123] Mutschler M., Paffrath T., Wolfl C., Probst C., Nienaber U., Schipper I.B., Bouillon B., Maegele M. (2014). The ATLS((R)) classification of hypovolaemic shock: A well established teaching tool on the edge?. Injury.

[124] Rossaint R., Bouillon B., Cerny V., Coats T.J., Duranteau J., Fernández-Mondéjar E., Hunt B.J., Komadina R., Nardi G., Neugebauer E. (2010). Management of bleeding following major trauma: An updated European guideline. Crit. Care.

[125] Simmons J.W., Pittet J.F., Pierce B. (2014). Trauma-induced coagulopathy. Curr. Anesthesiol. Rep..

[126] Gonzalez E., Moore E.E., Moore H.B. (2017). Management of Trauma-Induced Coagulopathy with Thrombelastography. Crit. Care Clin..

[127] Baksaas-Aasen K., Van Dieren S., Balvers K., Juffermans N.P., Næss P.A., Rourke C., Eaglestone S., Ostrowski S.R., Stensballe J., Stanworth S. (2019). Data-driven Development of ROTEM and TEG Algorithms for the Management of Trauma Hemorrhage: A Prospective Observational Multicenter Study. Ann. Surg..

[128] Gonzalez E., Moore E.E., Moore H.B., Chapman M.P., Chin T.L., Ghasabyan A., Wohlauer M.V., Barnett C.C., Bensard D.D., Biffl W.L. (2016). Goal-directed Hemostatic Resuscitation of Trauma-induced Coagulopathy: A Pragmatic Randomized Clinical Trial Comparing a Viscoelastic Assay to Conventional Coagulation Assays. Ann. Surg..

[129] Wikkelsø A., Wetterslev J., Møller A.M., Afshari A. (2016). Thromboelastography (TEG) or thromboelastometry (ROTEM) to monitor haemostatic treatment versus usual care in adults or children with bleeding. Cochrane Database Syst. Rev..

[130] Maegele M. (2021). The European Perspective on the Management of Acute Major Hemorrhage and Coagulopathy after Trauma: Summary of the 2019 Updated European Guideline. J. Clin. Med..

[131] Savioli G., Ceresa I.F., Maggioni P., Lava M., Ricevuti G., Manzoni F., Oddone E., Bressan M.A. (2020). Impact of ED Organization with a Holding Area and a Dedicated Team on the Adherence to International Guidelines for Patients with Acute Pulmonary Embolism: Experience of an Emergency Department Organized in Areas of Intensity of Care. Medicines.

[132] Savioli G., Ceresa I.F., Manzoni F., Ricevuti G., Bressan M.A., Oddone E. (2020). Role of a Brief Intensive Observation Area with a Dedicated Team of Doctors in the Management of Acute Heart Failure Patients: A Retrospective Observational Study. Medicina.

[133] Ceresa I.F., Savioli G., Angeli V., Novelli V., Muzzi A., Grugnetti G., Cobianchi L., Manzoni F., Klersy C., Lago P. (2020). Preparing for the Maximum Emergency with a Simulation: A Table-Top Test to Evaluate Bed Surge Capacity and Staff Compliance with Training. Open Access Emerg Med..

[134] Savioli G., Ceresa I.F., Novara E., Persiano T., Grulli F., Ricevuti G., Bressan M.A., Oddone E. (2021). Brief Intensive Observation areas in the management of acute heart failure in elderly patients leading to high stabilisation rate and less admissions. J. Gerontol. Geriatr..

[135] Neal M.D., Moore E.E., Walsh M., Thomas S., Callcut R.A., Kornblith L.Z., Schreiber M., Ekeh A.P., Singer A.J., Lottenberg L. (2020). A comparison between the TEG 6s and TEG 5000 analyzers to assess coagulation in trauma patients. J. Trauma Acute Care Surg..

[136] Spahn D.R. (2014). TEG(R)- or ROTEM(R)-based individualized goal-directed coagulation algorithms: Don’t wait–act now!. Crit. Care.

[137] Brenni M., Worn M., Bruesch M., Spahn D.R., Ganter M.T. (2010). Successful rotational thromboelastometry-guided treatment of traumatic haemorrhage, hyperfibrinolysis and coagulopathy. Acta Anaesthesiol. Scand..

[138] Kashuk J.L., Moore E.E., Johnson J.L., Haenel J., Wilson M., Moore J.B., Cothren C.C., Biffl W.L., Banerjee A., Sauaia A. (2008). Postinjury life threatening coagulopathy: Is 1:1 fresh frozen plasma:packed red blood cells the answer?. J. Trauma.

[139] Nienaber U., Innerhofer P., Westermann I., Schöchl H., Attal R., Breitkopf R., Maegele M. (2011). The impact of fresh frozen plasma vs coagulation factor concentrates on morbidity and mortality in trauma-associated haemorrhage and massive transfusion. Injury.

[140] Riskin D.J., Tsai T.C., Riskin L., Hernandez-Boussard T., Purtill M., Maggio P.M., Spain D.A., Brundage S.I. (2009). Massive transfusion protocols: The role of aggressive resuscitation versus product ratio in mortality reduction. J. Am. Coll. Surg..

[141] Schöchl H., Nienaber U., Hofer G., Voelckel W., Jambor C., Scharbert G., Kozek-Langenecker S., Solomon C. (2010). Goal-directed coagulation management of major trauma patients using thromboelastometry (ROTEM)-guided administration of fibrinogen concentrate and prothrombin complex concentrate. Crit. Care.

[142] Weber C.F., Gorlinger K., Meininger D., Herrmann E., Bingold T., Moritz A., Cohn L.H., Zacharowski K. (2012). Point-of-care testing: A prospective, randomized clinical trial of efficacy in coagulopathic cardiac surgery patients. Anesthesiology.

[143] Nakayama Y., Nakajima Y., Tanaka K.A., Sessler D.I., Maeda S., Iida J., Ogawa S., Mizobe T. (2015). Thromboelastometry-guided intraoperative haemostatic management reduces bleeding and red cell transfusion after paediatric cardiac surgery. Br. J. Anaesth..

[144] Karkouti K., McCluskey S.A., Callum J., Freedman J., Selby R., Timoumi T., Roy D., Rao V. (2015). Evaluation of a novel transfusion algorithm employing point-of-care coagulation assays in cardiac surgery: A retrospective cohort study with interrupted time-series analysis. Anesthesiology.

[145] Görlinger K., Dirkmann D., Hanke A.A., Kamler M., Kottenberg E., Thielmann M., Jakob H., Peters J. (2011). First-line therapy with coagulation factor concentrates combined with point-of-care coagulation testing is associated with decreased allogeneic blood transfusion in cardiovascular surgery: A retrospective, single-center cohort study. Anesthesiology.

[146] Theusinger O.M., Wanner G.A., Emmert M.Y., Billeter A., Eismon J., Seifert B., Simmen H.P., Spahn D.R., Baulig W. (2011). Hyperfibrinolysis diagnosed by rotational thromboelastometry (ROTEM) is associated with higher mortality in patients with severe trauma. Anesth. Analg..

[147] Levrat A., Gros A., Rugeri L., Inaba K., Floccard B., Negrier C., David J.S. (2008). Evaluation of rotation thrombelastography for the diagnosis of hyperfibrinolysis in trauma patients. Br. J. Anaesth..

[148] El-Menyar A., Ramzee A.F., Asim M., Di Somma S., Al-Thani H. (2020). Comparative analysis for the implication of serum cardiac troponin measurements by conventional versus high-sensitivity assays in patients with traumatic brain injury. Minerva Cardioangiol..

[149] El-Menyar A., Asim M., Ramzee A.F., Nabir S., Ahmed M.N., Al-Thani A., Al-Abdulmalek A., Al-Thani H. (2019). Bio-Shock Index: Proposal and Rationale for a New Predictive Tool for In-Hospital Mortality in Patients with Traumatic Brain Injury. World Neurosurg..

[150] Jackson M.R., Olson D.W., Beckett W.C., Olsen S.B., Robertson F.M. (1992). Abdominal vascular trauma: A review of 106 injuries. Am. Surg..

[151] Rossaint R., Bouillon B., Cerny V., Coats T.J., Duranteau J., Fernández-Mondéjar E., Filipescu D., Hunt B.J., Komadina R., Nardi G. (2016). The European guideline on management of major bleeding and coagulopathy following trauma: Fourth edition. Crit. Care.

[152] Johnson J.W., Gracias V.H., Schwab C.W., Reilly P.M., Kauder D.R., Shapiro M.B., Dabrowski G.P., Rotondo M.F. (2001). Evolution in damage control for exsanguinating penetrating abdominal injury. J. Trauma.

[153] Billy L.J., Amato J.J., Rich N.M. (1971). Aortic injuries in Vietnam. Surgery.

[154] American College of Surgeons (2018). Advanced Trauma Life Support (ATLS®).

[155] Derakhshanfar H., Vafaei A., Tabatabaey A., Noori S. (2017). Prevalence and Associated Factors of Acute Traumatic Coagulopathy; a Cross Sectional Study. Emergency.

[156] Campanella R., Guarnaccia L., Cordiglieri C., Trombetta E., Caroli M., Carrabba G., La Verde N., Rampini P., Gaudino C., Costa A. (2020). Tumor-Educated Platelets and Angiogenesis in Glioblastoma: Another Brick in the Wall for Novel Prognostic and Targetable Biomarkers, Changing the Vision from a Localized Tumor to a Systemic Pathology. Cells.

[157] Brohi K. (2009). Trauma induced coagulopathy. J. R. Army Med. Corps.

[158] Johansson P.I., Sorensen A.M., Perner A., Welling K.L., Wanscher M., Larsen C.F., Ostrowski S.R. (2011). Disseminated intravascular coagulation or acute coagulopathy of trauma shock early after trauma? An observational study. Crit. Care.

[159] Frith D., Brohi K. (2012). The pathophysiology of trauma-induced coagulopathy. Curr. Opin. Crit. Care.

[160] Bocci M.G., Nardi G., Veronesi G., Rondinelli M.B., Palma A., Fiore V., De Candia E., Bianchi M., Maresca M., Barelli R. (2019). Early coagulation support protocol: A valid approach in real-life management of major trauma patients. Results from two Italian centres. Injury.

[161] Cianci P., Fersini A., Tartaglia N., Altamura A., Lizzi V., Stoppino L.P., Macarini L., Ambrosi A., Neri V. (2016). Spleen assessment after laparoscopic transperitoneal left adrenalectomy: Preliminary results. Surg. Endosc..

[162] Cianci P., Fersini A., Tartaglia N., Ambrosi A., Neri V. (2016). Are there differences between the right and left laparoscopic adrenalectomy? Our experience. Ann. Ital. Chir..

[163] Cianci P., Tartaglia N., Altamura A., Di Lascia A., Fersini A., Neri V., Ambrosi A. (2018). Cervical Esophagotomy for Foreign Body Extraction: A Case Report and Extensive Literature Review of the Last 20 Years. Am. J. Case Rep..

[164] Di Lascia A., Tartaglia N., Fersini A., Petruzzelli F., Ambrosi A. (2018). Endoscopy for treating minor post-cholecystectomy biliary fistula A review of the literature. Ann. Ital. Chir..

[165] Tartaglia N., Petruzzelli F., Vovola F., Fersini A., Ambrosi A. (2019). Antegrade cholecystectomy before ligating the elements. A technique that reduces complications. Ann. Ital. Chir..

[166] Thorn S., Güting H., Maegele M., Gruen R.L., Mitra B. (2019). Early Identification of Acute Traumatic Coagulopathy Using Clinical Prediction Tools: A Systematic Review. Medicina.

[167] Curry N., Hopewell S., Doree C., Hyde C., Brohi K., Stanworth S. (2011). The acute management of trauma hemorrhage: A systematic review of randomized controlled trials. Crit. Care.

[168] Spahn D.R., Bouillon B., Cerny V., Duranteau J., Filipescu D., Hunt B.J., Komadina R., Maegele M., Nardi G., Riddez L. (2019). The European guideline on management of major bleeding and coagulopathy following trauma: Fifth edition. Crit. Care.

[169] Lucifero A.G., Luzzi S., Gragnaniello C., Savioli G., Tartaglia N., Ambrosi A. (2020). Hand-assisted laparoscopic vs. mini-laparot omy technique for ventriculoperitoneal shunt. A meta-analysis of three thousand patients. Ann. Ital. Chir..

[170] Savioli G., Ceresa I.F., Macedonio S., Gerosa S., Belliato M., Luzzi S., Lucifero A.G., Manzoni F., Ricevuti G., Bressan M.A. (2020). Major Trauma in Elderly Patients: Worse Mortality and Outcomes in An Italian Trauma Center. J. Emergencies Trauma Shock.

